# Chromosomal imbalances associated with carcinoma in situ and associated testicular germ cell tumours of adolescents and adults

**DOI:** 10.1054/bjoc.2001.1889

**Published:** 2001-07

**Authors:** B Summersgill, P Osin, Y-J Lu, R Huddart, J Shipley

**Affiliations:** Molecular Cytogenetics Team, Section of Molecular Carcinogenesis, Institute of Cancer Research, 15 Cotswold Road, Belmont, Sutton, Surrey, SM2 5NG, UK; Section of Radiotherapy, Institute of Cancer Research, 15 Cotswold Road, Belmont, Sutton, SM2 5NG, UK

**Keywords:** testicular cancer, carcinoma in situ, comparative genomic hybridization

## Abstract

Carcinoma in situ (CIS) or intratubular germ cell neoplasia is generally considered the precursor lesion of adult testicular germ cell tumours (TGCT). The chromosomal imbalances associated with CIS and the corresponding seminoma (SE) or nonseminoma (NS) have been determined by comparative genomic hybridization (CGH) analysis of microdissected material from seven cases. Significantly, the CIS showed no gain of 12p material whereas in the invasive components of all cases gain of 12p was found, in 2 cases associated with amplification of the 12p11.2–12.1 region. Interphase fluorescence in situ analysis was consistent with this and provided evidence for the i(12p) or 12p11.2–12.1 amplification in the SE and NS but not in the corresponding CIS. This suggests a role for these changes in progression of CIS to invasive testicular cancer or progression of the invasive disease. Other imbalances such as gain of material from chromosomes 1, 5, 7, 8, 12q and X and loss of material from chromosome 18 were frequently identified (> 40% of cases) in the CIS associated with both SE and NS as well as in the invasive components. Loss of material from chromosome 4 and 13 and gain of 2p were more frequently found in the invasive components. The results shed light on the genetic relationship between the non-invasive and invasive components of testicular cancer and the stage at which particular chromosomal changes may be important. © 2001 Cancer Research Campaign http://www.bjcancer.com


British Journal of Cancer (2001) 85(2), 213–219
© 2001 Cancer Research Campaign
doi: 10.1054/ bjoc.2001.1889, available online at http://www.idealibrary.com on IDEAL®

### Chromosomal imbalances associated with carcinoma in situ and associated testicular germ cell tumours of adolescents and adults

**B Summersgill[1], P Osin[1], Y-J Lu[1], R Huddart[2] and J Shipley[1]**

[1]Molecular Cytogenetics Team, Section of Molecular Carcinogenesis, Institute of Cancer Research, 15 Cotswold Road, Belmont, Sutton, Surrey, SM2 5NG, UK; [2]Section of Radiotherapy, Institute of Cancer Research, 15 Cotswold Road, Belmont, Sutton, Surrey, SM2 5NG, UK

**Summary** Carcinoma in situ (CIS) or intratubular germ cell neoplasia is generally considered the precursor lesion of adult testicular germ cell tumours (TGCT). The chromosomal imbalances associated with CIS and the corresponding seminoma (SE) or nonseminoma (NS) have been determined by comparative genomic hybridization (CGH) analysis of microdissected material from seven cases. Significantly, the CIS showed no gain of 12p material whereas in the invasive components of all cases gain of 12p was found, in 2 cases associated with amplification of the 12p11.2–12.1 region. Interphase fluorescence in situ analysis was consistent with this and provided evidence for the i(12p) or 12p11.2–12.1 amplification in the SE and NS but not in the corresponding CIS. This suggests a role for these changes in progression of CIS to invasive testicular cancer or progression of the invasive disease. Other imbalances such as gain of material from chromosomes 1, 5, 7, 8, 12q and X and loss of material from chromosome 18 were frequently identified (> 40% of cases) in the CIS associated with both SE and NS as well as in the invasive components. Loss of material from chromosome 4 and 13 and gain of 2p were more frequently found in the invasive components. The results shed light on the genetic relationship between the non-invasive and invasive components of testicular cancer and the stage at which particular chromosomal changes may be important. © 2001 Cancer Research Campaign http://www.bjcancer.com

**Keywords**: testicular cancer; carcinoma in situ; comparative genomic hybridization

Testicular germ cell tumours (TGCT) are the most common malignancy in young men. Histologically there are 2 main entities. These are the seminomas (SE) and the nonseminomas (NS). SE resemble primitive germ cells while NS show cellular differentiation features of embryonic and/or extra embryonic tissues. Some cases known as combined tumours show features of both SE and NS with the components either mixed or separated (Mostofi and Sobin, 1977).

The cellular origin of TGCT has been postulated variously to be either primordial/dysplastic germ cells or meiotic spermatocytes (Shakkebaek et al, 1987; de Jong et al, 1990; Chaganti and Houldsworth, 1998; Chaganti and Houldsworth, 2000). However, it is now generally accepted that carcinoma in situ (CIS), which are also known as intratubular germ cell neoplasia, are the precursor lesions for TGCT (Skakkebaeket et al, 1987). The CIS consist of cells with a uniform appearance similar to those which make up SE. All cases of TGCT have been shown to have gain of 12p material, most usually in the form of an i(12p) chromosome. In some cases the 12p11.2–12.1 subregion is involved in an amplification event (Suijkerbuijk et al, 1994; Mostert et al, 1996; Rao et al, 1998; Summersgill et al, 1998b). Karyotype and recent M-FISH analysis of cells derived from invasive GCT suggest that specific rearrangements probably do not involve breakpoints of significance and are likely to contribute to the tumour phenotype through resulting chromosome imbalances (van Echten et al, 1995; Summersgill et al 1998). Chromosomal and allelic imbalances are frequently found involving gain of material from chromosomes 7, 8, 12p, 21 and X and loss from chromosomes 4, 5, 11, 12q, 13, 18 and Y (de Jong et al, 1990; van Echten et al 1995; Sandberg et al, 1996; http://cgap.nci.nih.gov/ Chromosomes/ Mitelman).

Cytogenetic and molecular studies of SE and NS have shown considerable similarities in the abnormalities found suggesting a close developmental relationship between the groups (de Jong et al, 1990; Sandberg et al, 1996). 2 main pathways have been proposed to account for the origin of TGCT. SE and NS may arise through divergence at an early stage or according to a linear progression model where they both arise from a common CIS involving NS passing through a seminomatous stage (Mostofi, 1986; Oosterhuis et al, 1989; Sandberg et al, 1996; Changanti and Houldsworth, 2000). Genetic analysis of CIS and invasive testicular cancers has provided some insights. Despite the technical difficulties some limited cytogenetic data is available for CIS. The i(12p) has been identified in 2 out of 5 cases which have been karyotyped although in the other 3 cases rearrangements involving 12p and a number of unidentifiable chromosomes were found which could have resulted in additional 12p material (Vos et al, 1990; van Echten et al, 1995). A near tetraploid chromosome constitution is found in CIS associated with either SE and NS (de Jong et al, 1990; Dieckmann and Skakkebaek, 1999) and through restriction fragment length polymorphism analysis of 12q markers, formation of the i(12p) is suggested to occur after tetraploidisation (Geurts van Kessel et al, 1989). More recently, CGH analysis of CIS associated with single cases of SE and NS

*Received 10 November 2000*
*Revised 7 March 2001*
*Accepted 20 March 2001*

*Correspondence to:* J Shipley showed gain of 12p in one case (Looijenga et al, 2000). Interphase study of nuclei from semen samples from 10 patients with CIS have suggested that the i(12p) may be present in a few cases although further studies were recommended to clarify this point (Meng et al, 1998). The fact that the i(12p) appears to be present in some CIS has been interpreted to suggest that it may be associated with the genesis of these tumours (Changanti and Houldsworth, 2000).

Interphase FISH analysis using centromere probes for chromosomes 1, 12 and 15 has suggested genetic imbalances of these in CIS and the possibility that the chromosomal constitution of CIS associated with SE (CIS-SE) and NS (CIS-NS) are different (Looijenga et al, 1993). Recent loss of heterozygosity analysis for selected loci indicated common regions of loss in CIS and invasive SE and NS (Faulkner et al, 2000). Here we have sought to identify the chromosomal imbalances found in CIS and associated SE and NS using microdissection, universal DNA amplification and CGH analysis and interphase FISH analysis for 12p. This would address the issue of whether the CIS carry changes typical of their invasive counterparts.

### MATERIALS AND METHODS

### Tumour samples and microdissection

Clinical and histological data on the TGCT cases and CIS, SE and NS studied are summarized in Table 1. Lesions were classified according to Mostofi and Sobin (1977). Regions for microdissection were identified using 4 μm formalin-fixed paraffin-embedded sections stained with haemotoxylin and eosin (H&E) and mounted. Manual microdissection using drawn out glass rods was carried out on unmounted parallel 4 μm sections stained with H&E as previously described (Lu et al, 1998). A few regions of CIS were microdissected and pooled and single regions of SE or NS adjacent to these on the same section were collected. Frozen material from cases 19, 33, 32 and 46 have been previously analysed by CGH (Summersgill et al, 1998a, 1998b).

### CGH and digital image analysis

DNA was crudely extracted by boiling dewaxed, microdissected paraffin material and then subjected to PCR using degenerate oligonucleotide primers (DOP-PCR) (Telenius et al, 1992). Additional cycles of amplification were required in some cases to obtain visible amounts of DNA as assessed on an agarose gel. Direct labelling with Fluorescein-12-dUTP or Rhodamine-12-dUTP (FlouroGreen, FlouroRed, Amersham International plc, Amersham, UK) was carried out by incorporation during further rounds of DOP-PCR. For all CGH preparations, 250–500 ng of differentially labelled test and sex-matched control DNA from formalin-fixed, paraffin-embedded samples which had also been subjected to DOP-PCR, plus 15–25 μg of CotI DNA (BRL Gibco) were co-hybridized to normal denatured metaphases for 48 hours at 37˚C before washing and mounting in antifade with 0.1 μg ml$^{-1}$ 4, 6-diamidino-2-phenylindole (DAPI) as a counterstain. Images were captured using a cooled CCD camera (Photometrics) with software from Vysis, UK. CGH analysis was carried out as previously described using the same software package and independantly checked (Lu et al, 1998; Summersgill et al, 1998a, 1998b). At least 5 representative images were fully analysed and the results from these were studied separately and also combined to produce an average fluorescence ratio for each chromosome. The average normal ratio levels and their standard deviation were determined in CGH experiments using differentially labelled control DNA from paraffin-embedded material processed in the same way as the test samples and which had been subjected to DOP-PCR. A copy number change was indicated when the average fluorescence ratio corresponding to a sample lay outside the normal standard deviation. The average fluorescence ratios in control experiments plus and minus their standard deviation were found to be within the range 0.85–1.15. A copy number change was scored when the average fluorescence ratio was outside these limits and a ratio greater than 1.5 was scored as a higher level of gain or amplification.

**Table 1** Clinicopathological data and the material studied

| Case No. | Age in years at diagnosis | Histology | Material studied |
|---|---|---|---|
| 2 | 24 | SE | SE,CIS |
| 19 | 26 | SE | SE,CIS |
| 33 | 32 | SE | SE,CIS |
| 38 | 33 | SE | SE,CIS |
| 4 | 38 | SE+MTI | SE,CIS |
| 32 | 36 | NS,MTU | Emb.Ca,CIS |
| 46 | 29 | NS,MT | Emb.Ca,CIS |

SE, seminoma; NS, nonseminoma; MT malignant teratoma; MTI, malignant teratoma intermediate; MTU, malignant teratoma undifferentiated; Emb.Ca, embryonal carcinoma.

### Interphase FISH analysis

A probe for the 12 centromere and a YAC mapping to the minimal overlapping region of amplification (753f12, Whithead Institute http://www.genome.wi.mit.edu and supplied by UK MRC HGMP Resource Center) were used to identify the i(12p) and gain or amplification of 12p material. These probes were directly but differentially labelled, mixed and hybridized to standard 5 μm sections, using a microwave oven for denaturation, as previously described (Lu et al, 1999). The slides were then washed and mounted in antifade (Citifluor) with 0.1 μg ml$^{-1}$ DAPI. Parallel slides stained with H&E were used to help correlate the hybridization signals with cellular morphology. To locate CIS a digital vernier reading for the stage position was used relative to a feature recognisable using fluorescence microscopy. Images were collected using a Zeiss Axioplan microscope with appropriate filters, coupled to a cooled CCD camera (Photometrics, Tucson, AZ) and software from Vysis (UK Ltd). Several images from the same area but in different focal planes were taken in order to identify all the signals in the sections.

### RESULTS

Examples of CGH profiles for chromosome 12 are shown in Figure 1 for CIS and invasive components. The latter were determined to have either gain of 12p, (cases 2, 19, 33, 32 and 46) or amplification of the 12p11.2–12.1 region (cases 4 and 38). The results were consistent with interphase FISH analysis using the chromosome 12 markers described assuming the near tetraploid composition reported for CIS (de Jong et al, 1990; Dieckmann and Skakkebaek, 1999). Examples of interphase FISH for CIS and the corresponding invasive tumour are also shown in Figure 1. Figure 1B

 © 2001 Cancer Research Campaign

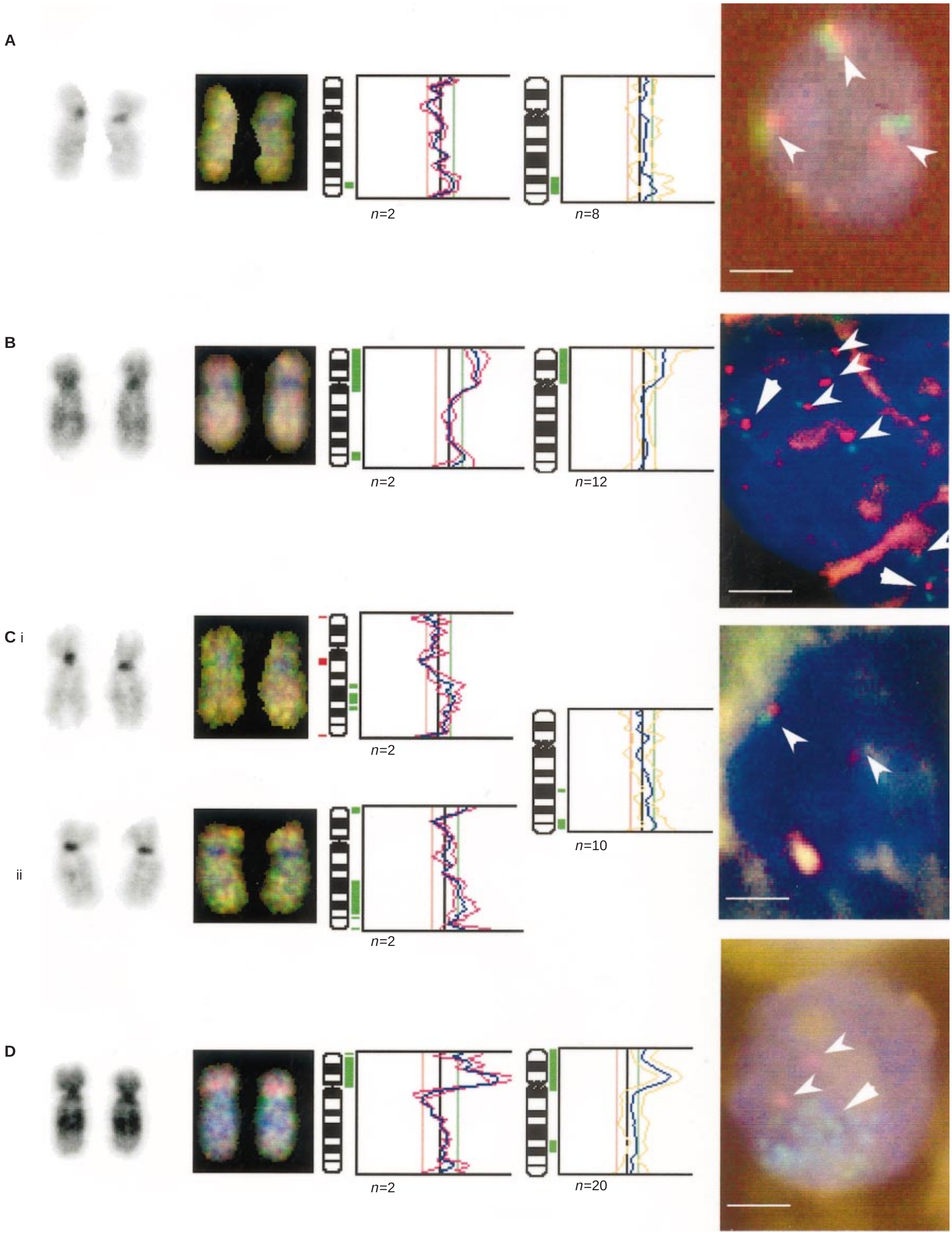

**Figure 1** Comparative genomic hybridization and interphase FISH analysis for chromosome 12 of CIS and associated SE and NS. (**A**) and (**C**) show the results for CIS for cases 2 and 38 respectively. (**B**) and (**D**) show the results for the SE, cases 2 and 38 respectively. Each panel shows the inverted DAPI banded images for chromosome 12 with the corresponding CGH images. These are followed by the CGH ratio profiles for these chromosomes. For (**C**) 2 examples are presented, **i.** showing no 12p gain and **ii.** showing gain of the 12p13 region. The average profile of all the chromosomes analysed for each sample is shown with *n* equal to the number of chromosomes analysed. The final image is a representative interphase FISH image of a nucleus from histopathologically defined regions (see Methods). The green signals are from the YAC 753f12 which lies within the smallest overlapping region of gain in the 12p11.2–12.1 region known to be amplified in some cases. The red signals correspond to the centromere of chromosome 12. The image of the interphase nucleus in (**A**) for the CIS shows 3 red green pairs of signals in the configuration found in a normal 12 chromosome (arrowed). As CIS cells have been shown to be near tetraploid (see introduction), this is consistent with the CGH profile. A nucleus from the associated SE is shown in (**B**) with several pairs of signals (arrowed) plus 2 examples consistent with i(12p) where 2 green signals flank the red centromere (triangle). (**C**) shows a nucleus from the CIS of case 2 with 2 clear pairs of red green signals and (**D**) shows evidence for multiple copies of the YAC (triangle) corresponding to amplification of the 12p11.2–12.1 region. The scale bar is equal to 5 μm.

shows the typical configuration of interphase signals expected for the presence of an i(12p) chromosome with the signal from the 12p YAC found either side of the centromere. None of the CIS were found to have detectable gain or amplification of 12p material by CGH analysis. However, some of the chromosome 12 CGH profiles for the CIS of case 38 indicated gain of the 12p13 region although this was not significant in the averaged profile (Figure 1C). The interphase FISH analysis for CIS produced reliable signals in between 5 and 15 cells and was not consistent with the presence of the i(12p) or amplification although cells were interpreted as having 2 and 3 copies of chromosome 12 in the presumed near tetraploid background (Figure 1). This was in contrast to the results of the associated SE and NS which all showed 12p gain in the form of an i(12p) or amplification of 12p material by both techniques. Other imbalances were identified by CGH analysis in CIS, SE and NS. These results are summarized in Figure 2. The results from the microdissected invasive disease for cases 19, 33, 32 and 46 were similar to those from previous CGH analysis of snap-frozen samples from these patients (Summersgill et al, 1998a,b).

### DISCUSSION

CIS is generally considered the precursor lesion of TGCT (Dieckmann and Skakkebaek, 1999). This study has defined the chromosomal imbalances associated with CIS and the associated invasive disease in 7 cases. 5 samples of invasive disease were of seminomatous histology and 2 samples were described as nonseminomas with features of embryonal carcinoma. All the invasive components showed gain of material derived from 12p. In 5 cases this was consistent with gain of the whole arm but in one SE and one NS amplification of the 12p11.2–12.1 region was identified. Significantly, the CGH analysis of the CIS did not detect gain of 12p material nor amplification of the 12p11.2–12.1 region in any of the cases. However, other imbalances were identified in the CIS some of which were in common with the associated invasive component.

Gain of 12p material in the SE and NS studied is consistent with previous karyotype and CGH analyses of tumour samples which have shown that this is most usually associated with an i(12p) (van Echten et al, 1995; Korn et al, 1996; Mostert et al, 1996; Summersgill et al, 1998a; http://cgap.nci.nih.gov/Chromosomes/Mitelman). Previous analyses have also shown that some cases are associated with amplification of a specific subregion at 12p11.2–12.1 (Suijkerbuijk et al, 1994; Mostert et al, 1996; Rao et al, 1998; Summersgill et al, 1998b). The absence of detectable 12p gain in the CIS cannot exclude the possibility that a small proportion of the cells within the tubules have an i(12p). The CGH analysis averages the imbalances present in the microdissected sample and the interphase FISH analysis could miss a few cells carrying this change. However, other imbalances in common with the invasive components were identified suggesting that the CGH approach would have detected 12p gain if it was present in most cells. Also, previous CGH analysis of frozen material (Summersgill et al, 1998a,b) and the analysis here of microdissected formalin-fixed paraffin-embedded material equivalent in size to CIS from cases 19, 33, 32 and 46 produced similar results and was consistent with our previous investigations validating this type of approach (Lu et al, 1998; Summersgill et al, 1998b).

The amplification events in 2 cases of TGCT were confirmed by interphase FISH analysis using a marker from the common overlapping region of amplification (Figure 1B) (Mostert et al, 1998; Roelofs et al, 2000; Goker and Shipley, unpublished data). Amplification was not detected by interphase FISH analysis in the corresponding CIS. This is consistent with recent interphase FISH analysis of 4 cases showing amplification in the invasive and microinvasive components but not in the CIS (Roelofs et al, 2000). Our data are consistent with relative gain or amplification of material from 12p being associated with progression of CIS to invasive testicular cancer or progression of the invasive disease.

In contrast to our data showing the absence of additional 12p material in CIS previous data have suggested that some CIS show i(12p) while others did not. Despite the technical difficulties some limited cytogenetic data are available for CIS. Whilst this has the advantage of analysing single cells, these dividing cells may not be representative of most cells in the lesion. One study showed that 1 out of 3 cases had 2 copies of i(12p) although other rearrangements involving 12p were identified as well as a number of unidentifiable chromosomes which could have resulted in additional 12p material (Vos et al, 1990). A second cytogenetic study showed i(12p) in both the CIS and corresponding invasive components and a second case in which it was not clear whether an i(12p) chromosome was present or not (van Echten et al, 1995). More recently, CGH analysis of material from 2 cases with CIS and associated SE or NS showed gain of 12p in both components of one case (Looijenga et al, 2000). Analysis of semen has suggested that some cases with CIS may have cells with i(12p) (Meng et al, 1998). A recent study presented 12p data only from CGH and interphase FISH analysis of various histological elements from 11 cases (Rosenberg et al, 2000). Gain of 12p material was not found in any CIS component. Taken together the available data indicate that gain of 12p material may arise as a late event in CIS and is associated with invasive disease.

The CGH analysis of CIS from case 38 presented here showed gain of the 12p13 region in some of the individual profiles although the avaerage profile did not indicate gain of 12p (Figure 1). In similar analysis of 2 cases, the noninvasive element of a NS showed a trend towards gain of the 12p13 region (Looijenga et al, 2000). The *CCND2* gene at 12p13 which is involved in regulating the G1–S cell cycle checkpoint has been suggested as a candidate for involvement in TGCT and has been shown to be expressed in CIS (Houldsworth et al, 1997). Analysis of primary TGCT and cell lines have also suggested that the 12p13 region may be important (Henegariu et al, 1998). Further analysis of CIS and other genes in this and other regions of 12p, particularly including the 12p11.2–p112.1 region amplified, is warranted.

Although 12p gain was not identified in the CIS studied here, other imbalances were frequently found, such as gain of material from chromosomes 1, 5, 7, 8, 12p and X and loss of material from chromosomes 18 ($>40\%$ of cases) (Figure 2). These changes were found in the CIS associated with both SE and NS (CIS-SE and CIS-NS) supporting a common pathway of development. Similar changes were also found in the invasive components themselves and have been frequently found in previous studies of SE and NS (van Echten et al, 1995; Korn et al, 1996; Mostert et al, 1996; Summersgill et al, 1998a; http://cgap.nci.nih.gov/Chromosomes/Mitelman). Loss of chromosome 4 was identified in 4 of the invasive TGCT and has been previously noted. However, this change was not seen in the corresponding CIS suggesting its association with invasive disease (Figure 2).

 © 2001 Cancer Research Campaign

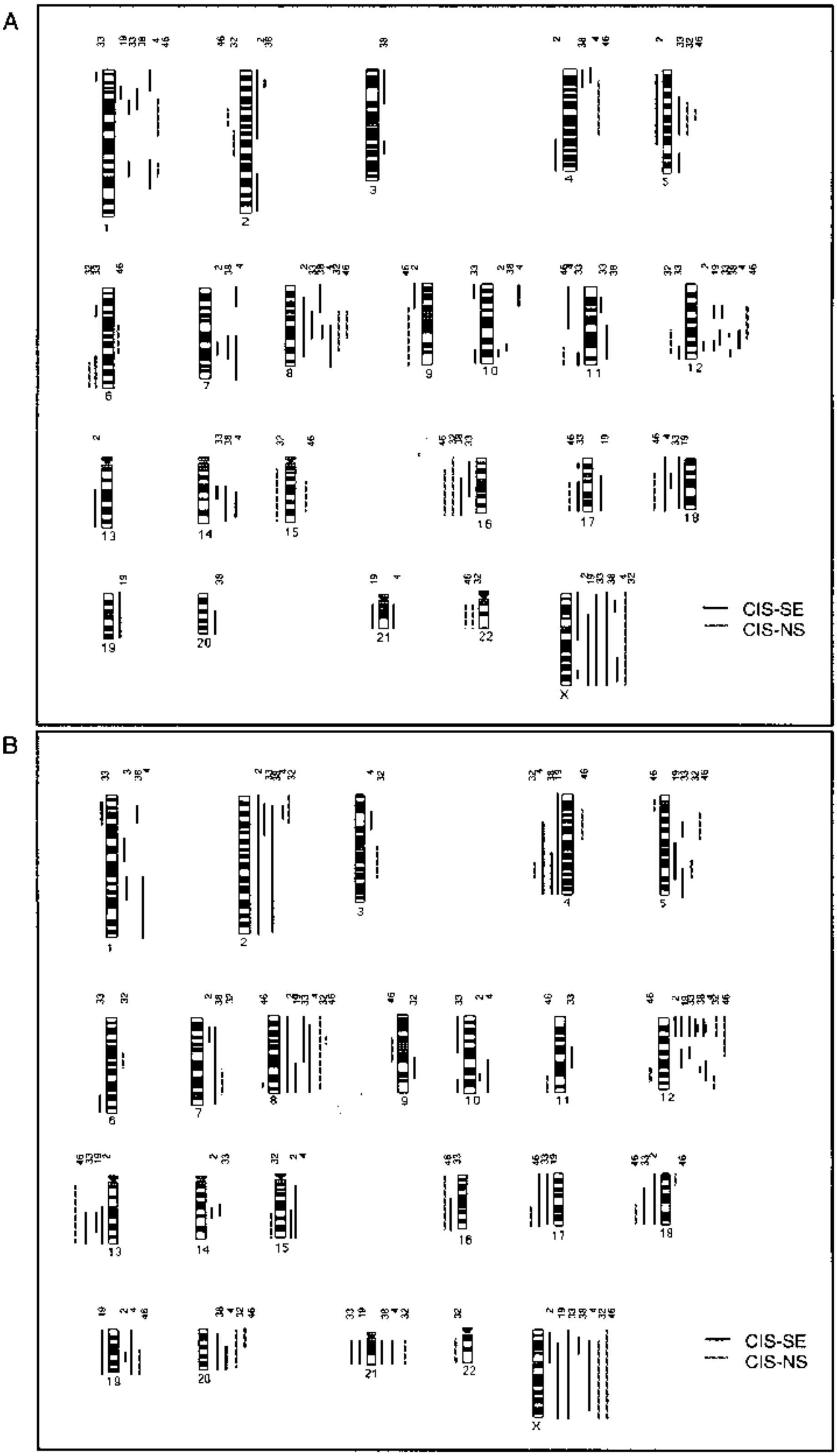

**Figure 2** Summary of the chromosomal imbalances associated with (**A**) CIS and (**B**) associated invasive SE and NS. Vertical lines on the right side of a chromosome represent gains of genetic material and vertical lines on the left side correspond to losses. Thick lines are indicative of a higher copy number change. Chromosomal imbalances in CIS associated with SE, CIS-SE, are represented by a solid line while imbalances in the CIS associated with NS, CIS-NS, are represented by the dashed line. The chromosomal imbalances in SE and NS are represented in (**B**) by solid and dashed lines, respectively. The case numbers are indicated on the top of the lines.

*© 2001 Cancer Research Campaign* 

Gain of 2p material was detected in 5 of the invasive TGCT but only in 2 CIS. Conversely, loss of material from chromosome 16 was more frequently found in the CIS. A similar situation has been shown in lobular carcinoma in situ of the breast involving chromosome 16 in which we suggest that loss has selective advantage for proliferation of cells within the ducts of the breast (Lu et al, 1998). A similar proliferative advantage relating to genes lost from chromosome 16 may operate within the seminiferous tubules that is no longer selected for in the invasive disease. Chromosome 16 loss is more frequently found in SE than NS and may be associated with the more seminomatous morphology which is associated with CIS. It has been postulated that all NS go through a seminomatous stage and the loss of 16 material in the CIS may reflect this (Oosterhuis et al, 1989).

Other chromosomal imbalances which are different between SE and NS have been previously noted (van Echten et al, 1995; Korn et al, 1996; Mostert et al, 1996; Summersgill et al, 1998a; http://cgap.nci.nih.gov/Chromosomes/Mitelman). These include an association of gain of 6q material and loss of 15 and 22 material with NS. One case of CIS-NS studied showed gain of 6q and this plus another case showed loss of 22. The latter case showing loss of chromosome 22 also had loss of chromosome 15 material in both components which is more typical of NS. These data suggest that the CIS associated with NS may already be committed to a nonseminomatous path of development. Previous interphase FISH analysis was also suggestive of this conclusion (Looijenga et al, 1993). However, more extensive investigations are required to identify the key determinants of NS and SE development.

### ACKNOWLEDGEMENTS

The authors would like to thank Rubin Wang and Osman Jafer for their technical support and helpful comments. This work was supported by the Cancer Research Campaign.

### REFERENCES

Chaganti R and Houldsworth J (1998) The cytogenetic theory of the pathogenesis of human adult male germ cell tumors. *Acta Pathol Microbiol Immunol Scand* **106**: 80–84

Chaganti R and Houldsworth J (2000) Genetics and biology of adult male germ cell tumours. *Cancer Res* **60**: 1475–1482

de Jong B, Oosterhuis JW, Castedo SMMJ, Vos AM and te Meerman GJ (1990) Pathogenesis of adult testicular germ cell tumors. A cytogenetic model. *Cancer Genet Cytogenet* **48**: 143–167

Dieckmann K and Skakkebaek N (1999) Carcinoma in situ of the testis: review of biological and clinical features. *Int J Cancer* **83**: 815–822

Faulkner S, Leigh D, Oosterhuis J, Roelofs H, Looijenga L and Friedlander M (2000) Allelic losses in carcinoma in situ and testicular germ cell tumors of adolescent and adults: evidence suggestive of the linear progression model. *Br J Cancer* **83**: 729–736

Geurts van Kessel A, van Drunen E, de Jong B, Oosterhuis J, A L and Mulder M (1989) Chromosome 12q heterozygosity is retained in i(12p)-positive germ cell tumor cells. *Cancer Genet Cytogenet* **40**: 129–134

Henegariu O, Vance G, Heiber D, Pera M and Heerema N (1998) Triple color FISH analysis of 12p amplification in testicular germ cell tumours using band-specific painting probes. *J Mol Med* **76**: 648–655

Houldsworth J, Reuter V, Bosl G and Chaganti R (1997) Aberrant expression of cyclin D2 is an early event in human male germ cell tumorigenesis. *Cell Growth Diff* **8**: 293–299

Korn W, Olde Weghuis D, Suijkerbuijk R, Schmidt U, Otto T, duManoir S, Geurts van Kessel A, Harstrick A, Seeber S and Becher R (1996) Detection of chromosomal DNA gains and losses in testicular germ cell tumours by comparative genomic hybridization. *Genes Chrom Cancer* **17**: 78–87

Looijenga L, Gillis A, van Putten W and Oosterhuis J (1993) In situ numeric analysis of centromeric regions of chromosomes 1, 12, and 15 of seminomas, nonseminomatous germ cell tumors, and carcinoma in situ of human testis. *Lab Invest* **68**: 211–219

Looijenga L, Rosenberg C, van Gurp R, Geelen E, van Echten-Arends J, de Jong B, Mostert M and Oosterhuis W (2000) Comparative genomic hybridization of microdissected samples from different stages of development of a seminoma and a non-seminoma. *J Pathol* **191**: 187–192

Lu Y-J, Osin P, Lakhani S, de Palma S, Gusterson B and Shipley J (1998) Comparative genomic hybridization analysis of lobular carcinoma in situ and atypical lobular hyperplasia and potential roles for gains and losses of genetic material in breast neoplasia. *Cancer Res* **58**: 4721–4727

Lu Y-J, Birdsall S, Summersgill B, Smedley D, Osin P, Fisher C and Shipley J (1999) Dual colour fluorescence in situ hybridisation to paraffin embedded samples to deduce the presence of the der (X)t(X;18)(p11.2;q11.2) and involvement of either the SSX1 or SSX2 gene: a diagnostic and prognostic aid for synovial sarcoma. *J Pathol* **87**: 490–496

Meng F, Zhou Y, Giwercman A, Skaaebaek N, Geurts van Kessel A and Suijkerbuijk R (1998) Fluorescence in situ hybridization analysis of chromosome 12 anomolies in semen cells from patients with carcinoma in situ of the testis. *J Pathol* **186**: 235–239

Mostert M, van de Pol M, Olde Weghuis D, Suijkerbuijk R, Geurts van Kessel A, van Echten J, Oosterhuis J and Looijenga L (1996) Comparative genomic hybridization of germ cell tumors of the adult testis: confirmation of karyotypic findings and identification of a 12p amplicon. *Cancer Genet Cytogenet* **89**: 146–152

Mostert M, Verkerk A, van de Pol M, Heighway J, Marynen P, Rosenberg C, Geurts van Kessel A, van Echten J, de Jong B, Oosterhuis J (1998) Identification of the critical region of 12p over-representation in testicular germ cell tumors of adolescents and adults. *Oncogene* **16**: 2617–2627

Mostofi F (1986) Pathology and germ cell tumors of the testis. *Cancer* **45**: 1735–154

Mostofi F and Sobin L (1977) International histological classification of testicular tumors (no. 6). In: International Histologic Classification of Tumors. Geneva: World Health Organization.

Oosterhuis J, Castedo S, B dJ, Cornelisse C, Dam A, Sleijfer D and Schraffordt-Koops H (1989) Ploidy of primary germ cell tumors of the testis: pathogenetic and clinical relevance. *Lab Invest* **60**: 14–20

Rao P, Houldsworth J, Palanisamy N, Murty V, Reuter R, Motzer R, Bosl G and Chaganti R (1998) Chromosomal amplification is associated with cisplatinum resistance of human male germ cell tumors. *Cancer Res* **58**: 4260–4263

Roelofs H, Mostert M, Pompe K, Zafarana G, van Oorschot M, van Gurp R, Gilles A, Stoop H, Beverloo B, Oosterhuis W (2000) Role of restricted 12p-amplification and RAS mutation in the development and clinical behaviour of human testicular germ cell tumors of adolescents and adults. *Am J Pathol* **157**: 1155–1166

Rosenberg C, Van Gurp R, Geelen E, Oosterhuis J and Looijenga L (2000) Overrepresentation of the short arm of chromosome 12 is related to invasive growth of human testicular seminomas and nonseminomas. *Oncogene* **19**: 5858–5862

Sandberg AA, Meloni AM and Suijkerbuijk RF (1996) Reviews of chromosome studies in urological tumors. III. Cytogenetics and genes in testicular tumors. *J Urol* **155**: 1531–1556

Skakkebaek N, Berthelsen J, Giwercman A and Muller J (1987) Carcinoma-in-situ of the testis: Possible origin from gonocytes, and precursor of all types of germ cell tumors except spermatocytoma. *Int J Androl* **10**: 19–28

Suijkerbuijk R, Sinke R, Olde Weghuis D, Roque L, Forus A, Stellink F, Siepman A, van de Kaa C, Soares J and Geurts van Kessel A (1994) Amplification of chromosome subregion 12p11.2-p12.1 in a metastasis of an i(12p)-negative seminoma: relationship to tumor progression? *Cancer Genet Cytogenet* **78**: 145–152

Summersgill B, Goker H, Weber-Hall S, Huddart R, Horwich A and Shipley J (1998a) Molecular cytogenetic analysis of adult testicular germ cell tumours and identification of regions of consensus copy number change. *Br J Cancer* **77**: 305–331

Summersgill B, Goker H, Osin P, Huddart R, Horwich A, Fisher C and Shipley J (1998b) Establishing the germ cell origin of undifferentiated tumours by identifying gain of 12p material using comparative genomic hybridisation analysis of paraffin embedded samples. *Diag Mol Path* **7**: 260–266

Summersgill B, Jafer O, Wang R, Goker H, Niculescu-Duvaz I, Huddart R and Shipley J (In press) Definition of chromosome aberrations in testicular germ cell tumor cell lines by 24 color karyotyping and complementary molecular cytogenetic analysis. *Cancer Genet Cytogenet*

Telenius H, Carter N, Bebb CE, Nordenskjold M, Ponder BAJ and Tunnacliffe A (1992) Degenerate oligonucleotide-primed PCR: general ampification of target DNA by a single degenerate primer. *Genomics* **13**: 718–725

van Echten J, Oosterhuis JW, Looijenga LHJ, van de Pol M, Wiersema J, Meerman GT, Schraffordt Koops H, D.T. S and de Jong B (1995a) No recurrent structural

 *© 2001 Cancer Research Campaign*

abnormalities apart from i(12p) in primary germ cell tumors of the adult testis. *Genes Chrom Cancer* **14**: 133–144

van Echten J, van Gurp J, Stoepker M, Looijenga L, de Jong B and Oosterhuis W (1995b) Cytogenetic evidence that carcinoma in situ is the precursor lesion for invasive testicular germ cell tumors. *Cancer Genet Cytogenet* **85**: 133–137

Vos A, Oosterhuis J, de Jong B, Buist J and Schraffordt Koops H (1990) Cytogenetics of carcinoma in situ of the testis. *Cancer Genet Cytogenet* **46**: 75–81

© 2001 Cancer Research Campaign
